# The immunological impact of revaccination in a hybrid-immune world

**DOI:** 10.3389/fimmu.2025.1588259

**Published:** 2025-06-09

**Authors:** Mary Bausch-Jurken, Galit Alter

**Affiliations:** ^1^ Moderna, Inc., Cambridge, MA, United States; ^2^ Ragon Institute of Mass General Brigham, Massachusetts Institute of Technology, Harvard University, Cambridge, MA, United States

**Keywords:** infectious diseases, COVID-19, hybrid immunity, immunology, public health

## Abstract

The global immune landscape of SARS-CoV-2 has progressively shifted from a naïve population several years ago to a population that possesses immunity to the virus through infection, vaccination, or a combination of both, known as hybrid immunity. Hybrid immunity offers a prolonged period of transmission-blocking activity, likely related to enhanced tissue-resident immunity, but also has been shown to be linked to broader humoral and cellular immune responses. Compared with vaccination or infection alone, the collective data have demonstrated that hybrid immunity offers enhanced protection against disease. Yet, despite the benefits of hybrid immunity, perpetual evolution of variants and the natural waning of immunity in vulnerable populations provides a strong rationale for revaccination. This article reviews the benefits of revaccination, including updating variant-specific immunity, bolstering humoral and cellular immune frequencies in those with hybrid immunity, and overcoming immune imprinting and enhancing effector mechanisms to raise surveillance and defense against the virus. As SARS-CoV-2 continues to evolve, updated booster vaccinations remain essential to enhance and sustain protection from disease by ensuring that the immune system is equipped to respond to contemporary strains, thereby reducing the impact of future outbreaks and mitigating the burden of COVID-19, especially among vulnerable populations.

## 1 Introduction

Several years after the first emergence of SARS-CoV-2 in 2019 (1), the global immune landscape has progressively evolved from a SARS-CoV-2–naïve population to one with hybrid immunity (2–5). Today, most of the world’s population has experienced a SARS-CoV-2 infection and/or received vaccination (2–4). The rapid and effective spread of SARS-CoV-2 is largely attributable to the unrelenting adaptation of the virus to population-level immunity, with an accumulation of escape mutations in the Spike antigen enabling successive surges of reinfections (6–8). While the majority of the world now possesses some level of immunity to the virus, these waves of reinfections continue to pose a real risk to vulnerable groups, including the aging population and individuals with compromised immunity, due to an inability of the immune system to adapt and block viruses with enhanced transmissibility or to respond with sufficient speed to contain the virus and prevent severe clinical outcomes (3, 6). In fact, older adults, those who are immunocompromised, and those with certain comorbid conditions continue to be hospitalized at the highest rates (9, 10), and COVID-19 continues to have a greater impact than influenza in terms of morbidity and mortality in the United States (11). Moreover, in the absence of robust immunity, these vulnerable populations are susceptible to post-acute sequelae, such as long COVID or post-COVID conditions, resulting in increased health burden and the risk of mortality (12, 13). Yet, the public health challenge is further complicated by the fact that the circulation of SARS-CoV-2 does not follow typical seasonality, but instead new variants emerge unpredictably throughout the year, rendering it difficult to time vaccine updates (14, 15).

Compared with vaccination or infection alone, emerging data have clearly demonstrated the superior level of protection conferred by the combination of vaccination and infection, termed hybrid immunity (5). Specifically, hybrid immunity offers a prolonged period of transmission-blocking activity (16, 17), likely related to enhanced tissue-resident immunity (18, 19), but also has been shown to be linked to broader humoral (20–22) and cellular immune responses (17, 23, 24). However, even in the setting of hybrid immunity, vulnerable populations continue to suffer from more severe disease and death, potentially related to reduced durability, compromised or senescent cellular immunity, or compromised capacity to elicit neutralizing antibodies in response to SARS-CoV-2 variants (25). SARS-CoV-2 variants are genetically mutated versions of the original virus that may have differing viral features, and are defined according to their lineages based on the genetic sequence of the Spike protein (26). Since the original SARS-CoV-2 was detected in 2019 (1), several variants have emerged, such as Alpha, Delta, and Omicron, each with varying degrees of transmissibility and disease severity (27). Development of first-generation COVID-19 vaccines targeting the original SARS-CoV-2 were proven to be effective, with subsequent development of variant-targeting vaccines as a response to manage emerging variants (28). Vaccination with the current COVID-19 seasonal vaccine provides an opportunity to not only bolster titers and effector cell frequencies, but also to reeducate and update the adaptive immune system to respond to the to the updated variant-focused vaccine (29–31). These more recently primed and educated immune responses create a higher barrier of immunity within these vulnerable populations and have the potential to reduce the risk of acute and post-acute COVID-19 events (32–35).

This review focuses on real-world evidence as well as the mechanisms of protection afforded by revaccination in individuals with hybrid immunity. We aim to explore and inform on the strategic use of vaccination to boost immunity and increase the window of protection, particularly among populations at higher risk for COVID-19. We also place this information in the context of subsequent protection against SARS-CoV-2 variants, particularly during multiple waves of omicron variants given the recent global recommendations for monovalent variant-updated vaccines (36).

## 2 Updating immunity to overcome imprinting

In October 2021, prior to the emergence of the highly transmissible omicron variant, global SARS-CoV-2 seroprevalence was estimated at 67% (37). SARS-CoV-2 seroprevalence in the United States has been largely attributed to vaccination (38). However, the steep increase in infections following the emergence of omicron in November 2021, due in part to the ineffectiveness of previous infection–induced immunity to protect against omicron, and expanded vaccine coverage led to increased vaccine- and infection-induced seroprevalence of >90%, with increases in hybrid immunity of 51%-60% by mid-2022 (37, 39–46). While it is difficult to determine the cause of seroprevalence at a population level, following repeated waves of global omicron sublineage evolution and transmission, it has been estimated that upwards of 90% of the globe has detectable SARS-CoV-2 antibodies from infection, vaccination, or hybrid immunity (46–49). The extraordinary speed of global transmission events coupled with the unprecedented speed of vaccine development, updating, and deployment has created remarkable heterogeneity in the frequency of vaccine and infection events across the population (40, 43, 47, 48, 50). However, emerging data suggest that the sequence and type of immune exposures substantially influence the quality of the immune response. For instance, the timing of vaccination following SARS-CoV-2 infection may influence post-vaccine IgG levels in hybrid immune individuals (51).

As SARS-CoV-2 continues to evolve, selective pressure through host immune responses can influence pathogen evolution to evade preexisting immunity (52). In the evolutionary arms race, the immune system rapidly adapts to respond to newly evolving variants, but often is heavily biased by preexisting memory B-cell specificities, also known as immune imprinting, that prevents the generation of truly *de novo* responses that find novel means to respond to evolving variants (53). This bias is due to the existence of a population of memory B cells that have high affinity and specificity to the initial strain, that compete aggressively for antigen in the germinal centers, preventing naïve B cells from competing for signals to proliferate and expand (53).

Despite the evolution of novel variants of concern and the inclusion of updated sequences in vaccines, both revaccination and reinfection result in the recall of the original virus-specific response to which an individual was first exposed (53). This imprinting manifests due to the rapid and preferential binding of preexisting SARS-CoV-2–specific B cells to shared epitopes on the circulating and original strain to which the individual was exposed (53). However, the non-shared, or escape, mutations found on the contemporaneous strain represent the critical immune targets for control, clearance, and future immunity against the current variant (54). Reinfection naturally stimulates evolution of the immune response to the circulating virus (55). However, for vulnerable populations that are susceptible to potential complications of infection, vaccination with updated strains represents a key approach to rapidly update and educate the immune response to adapt to respond to the evolving viral variants.

The importance of overcoming imprinting was clearly illustrated following the evolution of the delta and then omicron SARS-CoV-2 lineages (53). While vaccination with the original antigenic sequence conferred protection against delta variant infection, protection against omicron was reduced, and neutralizing antibody titers waned more quickly (56). This differential protection across the variants of concern was likely related to the higher conservation of delta to the original strain, resulting in more cross-reactive antibodies from the original Wuhan-based vaccine with the ability to neutralize delta compared with the more distant and less cross-reactive omicron (57–62). The critical importance of sequence degree of similarity between the variants became increasingly evident as reinfection rates were estimated to have increased from alpha-dominated periods (0.57, 95% confidence interval [CI]: 0.28–0.94]), through delta (1.25, 95% CI: 0.97–1.55), and through the initial wave of the highly divergent omicron (3.31, 95% CI: 1.15–6.53) (63). Of note, while comparing rates of re-infection across variants, the likelihood of exposure to the virus due to masking, social distancing, quarantining, etc. should be considered (64). The efficacy of infection-induced immunity alone against reinfection was estimated to be 65% (incidence rate ratio [IRR] = 0.35, 95% CI: 0.26–0.47), with the pooled IRRs for the alpha (IRR = 0.11), delta (IRR = 0.19), and omicron (IRR = 0.61) variants indicating progressively lower effectiveness (65). Despite widespread global immunity, the continued evolution of omicron subvariants JN.1, KP.2, and KP.3 subverted previous vaccine- and infection-induced immunity, necessitating updating the monovalent XBB.1.5-targeting vaccine.

Although genetic evolution occurs across the entire viral genome, continual changes in the receptor binding domain (RBD) of the Spike protein lead to evasion of population-level immunity to previous strains and are particularly relevant for vaccine-induced immunity (8, 36, 66). However, beyond their ability to evade preexisting humoral immune responses, several mutations, including those in the XBB variant and more recently in the KP sublineages of omicron, also exhibit increased transmissibility compared with SARS-CoV-2 variants that emerged during earlier phases of the pandemic (67, 68). This increased transmissibility has led to widespread waves of global infection (69). Yet, in the wake of these waves of infection and despite reduced neutralization of new variants, COVID-19 vaccination provided protection against severe disease and was associated with greater point estimates of protection against hospitalization among cases with XBB/XBB.1.5 versus non-XBB/XBB.1.5 cases (52). The XBB/XBB.1.5 lineage was more sensitive to immune responses triggered by vaccination than to those triggered by prior infection with pre-omicron or BA.4/BA.5 (52). However, updates to vaccine composition resulted in approximately 60% increased protection against infection caused by XBB.1.5, the variant targeted by the monovalent mRNA vaccine. The protection against infection provided by the monovalent XBB.1.5–targeting vaccine was only 49% against the emerging JN.1 variant (70), highlighting the critical importance of updating immunity to the more contemporaneously circulating variant. As such, the World Health Organization (WHO) recommended the inclusion of JN.1 antigen in vaccines to enhance protection against the JN.1 variant (71). Yet, JN.1 is now being replaced by JN.1 subvariants with mutations in the Spike protein, including FLiRT variants, KP.2, KP.3, and LB.1, which may have an even higher viral fitness (49). KP.2 has shown an almost 3-fold resistance to neutralization following XBB.1.5 vaccination and almost 2-fold resistance following previous infection (72). For the 2024–25 COVID-19 vaccine update, the US Food and Drug Administration (FDA) recommended including the KP.2 strain, intended to expose the immune system to the most recent dominant circulating variant to mount a variant-specific immune response (73). Particularly in vulnerable populations, updated vaccination may critically promote enhanced antibody titers to highly variable regions of the viral Spike glycoprotein, replenish effector T cell numbers, and update memory B cell clonal repertoires, enabling these cells to respond more effectively upon encounter with the next viral variant (31, 74).

Vaccination has been shown to provide protection from infection and enhance protection against illness in previously infected individuals (75). Conversely, in New Zealand, where vaccination rates were high with low levels of infection, the omicron wave early in 2022 led to nearly 24,000 daily cases and significant increases in hospitalization and intensive care unit admission, arguing that a combination of both infection and vaccination provided enhanced protection against severe disease (76). Omicron infected large swaths of the population globally, resulting primarily in upper respiratory disease (77, 78); however, the lack of concomitant increases in hospitalizations in vaccinated people (79) demonstrated the impact of vaccine-induced immunity against severe outcomes. In contrast, unvaccinated individuals in Hong Kong experienced high rates of hospitalization, severe disease, and death (80), highlighting the pathogenicity of omicron in non-immune populations. Moreover, additional revaccination with a fourth mRNA vaccine dose resulted in enhanced relative vaccine effectiveness against severe COVID-19 in adults aged ≥40 years, irrespective of infection history (81). The precise mechanism by which revaccination confers maximal protection against infection and disease in hybrid-immune individuals is likely via both quantitative and qualitative changes to the neutralizing/non-neutralizing antibody and adaptive cellular SARS-CoV-2–specific responses both peripherally and at mucosal barriers responsible for maximal immunity against this evolving pathogen. An overview of the features of SARS-CoV-2 infection-induced immunity, vaccination-induced immunity, and hybrid immunity is presented in **Table 1**.

**Table 1:** Comparative features of SARS-CoV-2 immunity types.

Immune Effectors	Infection	Vaccination	Hybrid Immunity
Neutralizing antibodies	+	++	+++
Fc-effector antibodies	++	+	+++
B-cell frequencies	+	++	+++
CD4+ T-cell responses	+	++	+++
CD8+ T-cell responses	++	-/+	+++
Mucosal immune responses	++	-/+	+++

"+" → *Low level immune response*

"++" → *Moderate level immune response*

"+++" → *High level immune response*

"++++" → *Very high or enhanced immune response*

"-/+" → *Variable or inconsistent response*

"-" or "---" → *Absent or no detectable immune response.*

### 2.1 Revaccination as a mechanism to update neutralizing antibody responses

**Figure 1:**
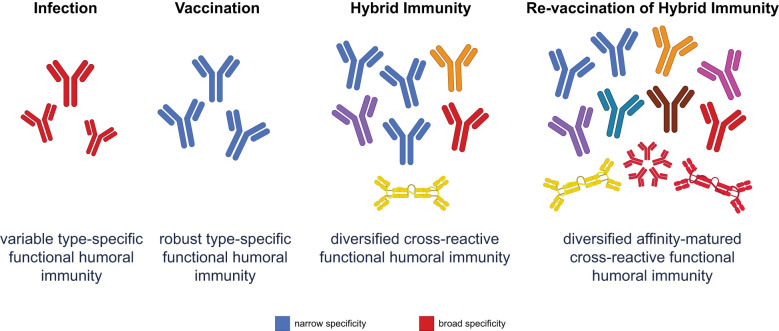
Functional humoral immunity varies across different immune scenarios. Infection produces a variable response with broad specificity, but limited affinity maturation. Vaccination elicits robust and specific antibodies, but narrowly focused protection. While hybrid immunity combines the strengths of both infection and vaccination, revaccination of hybrid-immune individuals further diversifies and matures the humoral response, leading to affinity-matured, highly cross-reactive antibodies. This approach optimally updates the immune repertoire to respond effectively to emerging variants, especially through improved mucosal and systemic functional immunity.

A significant number of studies have noted the critical immune synergy created by combined natural and vaccine-mediated protection in hybrid immunity, related to: broader proteomic coverage of humoral and cellular immune responses (74), improved breadth and magnitude of the neutralizing antibody response (51), enhanced B-cell affinity maturation (82, 83), shift in humoral immunodominance to more conserved regions of the Spike antigen (84, 85), and mucosal immune induction (86). As such, hybrid immunity allows for a broader, more diverse, and mucosally enriched humoral immune response compared with immunity from vaccination or infection alone (20, 24) (**Figure 1**). Specifically, vaccination alone induces highly potent but narrowly focused neutralizing antibody responses due to the stabilized presentation of the Spike antigen following COVID-19 vaccination (87). These responses are largely focused on the immunodominant RBD domain of the Spike antigen (88, 89), the primary region involved in attachment to the host angiotensin-converting enzyme 2 (ACE-2) receptor (90). However, infection alone or the combination of infection and vaccination results in an expansion of the humoral immune response to additional domains of the Spike antigen, including enhanced responses to the less mutable N-terminal (NTD) and S2 domains of the Spike antigen (85, 91). Despite their lower potency, expanded S2 responses have been shown to exhibit enhanced breadth of coverage across sarbecoviruses, and these findings may guide effective vaccine strategies for protection against evolving variants (92).

Critically, revaccination drives rapid and highly productive evolution of RBD-specific antibodies away from historical variants (93, 94) (**Figure 1**). Early revaccination data suggested that inclusion of the ancestral strain, in a combination vaccine, resulted in the continued selection and expansion of poorly cross-reactive, previously primed, B-cell responses (95, 96). Conversely, revaccination with updated variant vaccines only, ensured the evolution of the previously primed response towards the newer variant (97). However, the combination of infection and revaccination clearly illustrated the greatest increase in breadth of neutralization (98). The evolution of enhanced breadth of neutralization following breakthrough infection likely arose due to a combination of an updated Spike antigen, multiple structural presentations of the antigen on a virus or virally infected cell, the additional innate immune signals that are induced following infection (98), and potentially expanded T-cell helper signals. Together, these factors drive enhanced B-cell selection in the setting of hybrid immunity. However, upon revaccination, recall of these previously vaccine- and infection-primed B cells expands robustly, leveraging the rich preprogrammed memory B cells that can now adapt rapidly and robustly to potentially new viral variant sequences (98).

**Figure 2:**
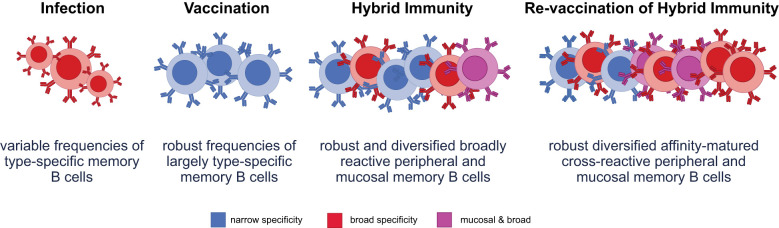
Varying immune exposures differentially characterize functional B-cell responses. Infection produces variable frequencies of memory B cells, with some broad reactivity due to exposure to diverse viral antigens, but typically limited by lower somatic hypermutation and affinity maturation. Vaccination induces robust frequencies of memory B cells that are largely type-specific and focused on the vaccine antigen, with limited breadth and minimal mucosal targeting. Hybrid immunity results in a robust and diversified memory B-cell pool, encompassing both peripheral and mucosal compartments. This broad reactivity enables improved protection across variants and sites of viral entry. Revaccination of hybrid-immune individuals enhances the diversity, affinity maturation, and mucosal targeting of memory B cells, producing a highly cross-reactive and protective memory B-cell repertoire optimized for variant recognition and clearance.

Mounting evidence suggests that hybrid immunity results in the presence of higher frequencies of memory B cells compared with infection or vaccination alone (22, 82); the B cells are primed for a more robust response upon revaccination (22) (**Figure 2**). Moreover, deeper B-cell clonal repertoire analyses illustrated that hybrid immunity resulted in the recruitment of broader, more affinity-matured, clonal repertoires that are resilient to evolving variants (99, 100). Specifically, RBD-specific neutralizing antibodies isolated from unvaccinated individuals following infection tend to show little or no somatic mutation, whereas antibodies following three doses of mRNA vaccine or breakthrough infection show high levels of somatic mutation, leading to greater cross-neutralization (101). These broader, richer, clonal repertoires still vary across hybrid populations, depending on the variant that caused infection. However, hybrid immunity likely provides a broader repertoire upon which novel variant boosting may help adapt immune responses more contemporaneously (93, 94, 99, 100).

Revaccination in a hybrid-immune individual furthermore is likely to recall memory B-cell responses that were programmed at the mucosal barrier in the setting of previous infection (102). Specifically, while vaccination alone does not elicit high immunoglobulin A (IgA) titers that are critical for mucosal protection, infection elicits high levels of systemic and mucosal IgA responses (103). Notably, revaccination elicited higher titers of IgG and IgA in saliva compared with primary immunization (104), arguing that revaccination has the capacity to bolster mucosal humoral immunity. Furthermore, hybrid immunity was associated with an enrichment of mucosal B-cell responses (MBCs) and tissue-resident CD4 and CD8 T-cell responses in the bronchioalveolar lavage (BAL) not observed in individuals that had only received a vaccine (105).

Studies have shown the cross-recognition potential of memory B cells primed by vaccination alone, particularly those exposed initially to the ancestral Wuhan-Hu-1 strain. In vaccine-only individuals, memory B cells elicited by the original mRNA vaccines retain the ability to recognize and respond to diverse SARS-CoV-2 variants, though with reduced breadth and potency compared with those with hybrid immunity (83, 106). The effect of re-vaccination using either monovalent (Wuhan-Hu-1) or bivalent (Wuhan-Hu-1 + Omicron BA.4/5) mRNA boosters has been shown to expand memory B cell breadth (107). Importantly, individuals with hybrid immunity demonstrate superior somatic hypermutation and affinity maturation in memory B cells, with improved recognition of divergent Spike proteins (108, 109). This evolution of the B cell response is particularly relevant in immunocompromised populations, where both the magnitude and quality of vaccine-induced memory B cells are often impaired.

### 2.2 Revaccination as a mechanism to drive non-neutralizing antibodies

Beyond the ability of highly affinity-matured antibodies to neutralize the virus, emerging data suggest that additional properties of antibodies may contribute to attenuation of disease. Specifically, beyond their ability to block infection, once complexed with a virus or virally infected cell, antibodies are able to rapidly interact with Fc-receptors found on all innate immune cells and drive rapid clearance or destruction of the complex (110). Importantly, naturally produced non-neutralizing antibody functions during infection have been linked to protection against severe disease and death (111). Additionally, convalescent plasma with the ability to drive antibody-mediated cytotoxicity was associated with protection against disease (112), and therapeutic monoclonal antibodies targeting the Spike protein depended on the ability to recruit the innate immune system via Fc-receptors to provide protection against disease (110). Interestingly, while these non-neutralizing antibody functions are induced by vaccination and infection, hybrid immunity has been shown to bolster these responses (113).

Unlike neutralizing antibodies that must target sites involved in host-cell binding or fusion mechanisms, non-neutralizing antibodies can target the whole surface of the Spike antigen (114). Both vaccination and infection have the potential to expand antibody function and responses across the Spike protein; however, infection-induced non-neutralizing antibodies may target a broader array of Spike antigen presentation states (pre- and post-fusion) as well as other virally encoded antigens (110, 115). Moreover, hybrid immunity has the potential to benefit from both vaccine- and infection-associated immune programming, inducing functional antibodies focused on an array of Spike antigen presentation states, programmed to elicit diverse antibody effector functions due to priming/boosting across inductive sites (22). Additionally, because non-neutralizing antibodies can target epitopes outside of the RBD, emerging data suggest that non-neutralizing functional antibodies are more resilient (85, 111, 113, 116), targeting less mutable segments of the Spike antigen. Thus, revaccination has the capacity to rapidly recall and boost variant-resilient, highly functional, humoral immune responses.

As mentioned above, revaccination in hybrid-immune populations is associated with the induction of broader antibody isotypes, including IgA, due to preexisting priming within mucosal inductive sites (117–122). Within mucosal tissues, IgA typically dimerizes, forming a quadrivalent molecule, able to bind incoming virus with higher avidity, which may enable IgA to continue to neutralize even in the setting of viral evolution (123, 124). Moreover, monomeric IgA may also interact with neutrophils that may be rapidly recruited to the mucosal barrier upon infection and drive rapid opsonophagocytosis of the virus (125–128). The importance of IgA responses to protection against COVID-19 was illustrated among healthcare workers in Barcelona, where higher IgA responses were observed among those with hybrid immunity compared with vaccination alone (129). Additionally, higher levels of IgG and IgA antibodies were shown to be a correlate of protection against breakthrough infection following revaccination (129, 130). While IgA responses in the nasal washes are higher in individuals following infection compared with vaccination, significantly higher levels of IgA and IgG are detectable in the nasal washes following boosting in individuals with hybrid immunity, with IgA antibodies contributing dominantly to neutralization in the mucosa (103).

Collectively, revaccination of hybrid-immune individuals leverages key humoral immune features that are likely key for protection against infection and disease: rapid affinity maturation and clonal diversification, enabling the rapid adaptation and updating of the humoral immune response for the most current viral variant (120, 131, 132), and expansion of functional humoral immunity to the whole surface of the Spike antigen, resulting in a highly resilient immune response able to rapidly capture and clear the infection (22). Thus, boosting of hybrid-immune B-cell repertoires is likely able to elicit more contemporaneously adapted immune responses at the mucosal barrier that may be essential to protect those most vulnerable in our population.

### 2.3 Revaccination as a mechanism to bolster T-cell immunity

**Figure 3:**
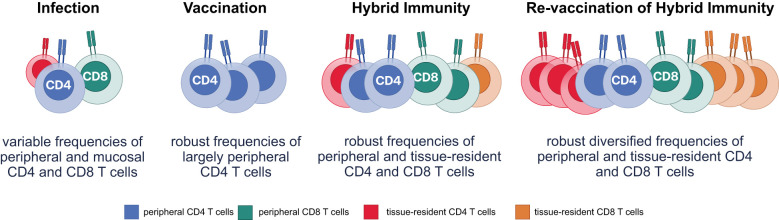
The landscape of functional T-cell immunity is shaped by varying immune scenarios. T-cell responses (CD4 and CD8) differ in frequency and localization across immune scenarios, impacting protection at peripheral and mucosal sites. Infection generates variable frequencies of peripheral and mucosal CD4 and CD8 T cells, influenced by the severity and location of infection, with limited consistency in tissue-resident T-cell populations. Vaccination induces robust frequencies of largely peripheral CD4 T cells, with limited impact on CD8 or tissue-resident T cells, providing systemic protection but minimal mucosal immunity. Hybrid immunity combines features of infection and vaccination, resulting in robust frequencies of both peripheral and tissue-resident CD4 and CD8 T cells, enhancing protection at mucosal sites and against reinfection. Revaccination of hybrid-immune individuals further diversifies and amplifies frequencies of both peripheral and tissue-resident CD4 and CD8 T cells, driving optimal immune surveillance and response at systemic and mucosal sites.

As novel variants arose, protection against hospitalization and death in vaccinated populations also pointed to a potential role for T cells as key correlates of protective immunity against COVID-19 disease (133). Along the same lines, early in the pandemic, studies highlighted an association between preexisting cross-reactive human coronavirus T cells and reduced risk of severe COVID-19 disease (134–137), due to conserved T-cell targets across previously circulating coronaviruses and SARS-CoV-2 (138). Moreover, the fact that vaccination provided benefit from severe disease and death in individuals lacking B cells due to treatments for autoimmunity or malignancies (111, 139) and was demonstrated to be effective in individuals with inborn errors of B-cell development (e.g., X-linked agammaglobulinemia) (140), further highlights the importance of T-cell immunity in disease attenuation (141, 142). Unlike neutralizing antibodies, vaccine-induced T-cell responses have exhibited remarkable resilience across variants (143–146), demonstrating persistent recognition of variant sequences (138). In the setting of hybrid immunity, T-cell–mediated immunity was characterized by detectable viral proteome-wide T-cell responses (147–149), broader clonal composition (150, 151), enhanced cytotoxic CD8 T-cell responses (150), and presence of T cells at mucosal sites (18) (**Figure 3**). Revaccination resulted in increased expansions of both CD8 and CD4 T-cell receptor (TCR) clonal repertoires and large numbers of CD8 T-cell responses (117–121), arming the immune system with a diverse and rich repertoire of T cells primed for antiviral activity upon viral re-exposure.

Importantly, after priming or revaccination, T-cell immune responses reach maximal numbers of both memory and effector cells. However, over time, terminally differentiated effector cells contract, resulting in the persistence of largely central memory T cells (152). Thus, while T-cell responses appear to persist over time following vaccination, infection, as well as in the setting of hybrid immunity (153), effector cell responses contract in all populations, resulting in the circulation of only memory T cells, and not armed effector cells ready to respond immediately upon reinfection. Activation of memory T cells occurs in response to active viral replication or following vaccination. However, the speed of activation and conversion of memory, also known as anamnestic immunity, is a critical determinant of the ability of T cells to control and clear infection (154, 155). Due to age- or inflammation-associated senescence, anamnestic immunity is dampened in older and immunocompromised individuals (156, 157). Instead, revaccination is able to bolster T-cell numbers, and particularly effector cells, ready to act upon encounter with a virally infected cell, which may be of the utmost importance in vulnerable populations that exhibit delayed proliferative kinetics and/or require higher frequencies of T cells due to compromised humoral immune responses (158). Within the cellular arm of the immune response, cytotoxic T cells (CD8 T cells) play a role in viral control and clearance by directly interacting with SARS-CoV-2–infected cells to suppress viral replication or kill the infected cells (159). In contrast, CD4 T cells play diverse roles, as helpers for B- and T-cell programming (159), but can also differentiate into cytotoxic cells that are induced at higher levels with hybrid immunity and are linked to protection against symptomatic disease (22). Importantly, among the CD4 T-cell subpopulations, follicular helper (Tfh) subpopulations play a key role in promoting effective humoral immune responses (160, 161). While vaccination alone was shown to elicit robust SARS-CoV-2 Spike-specific CD4 T-helper responses and low-level CD8 T-cell immunity (162–164), infection induces enhanced CD8 T-cell immunity (150). This observation argues that viral infection may promote enhanced cytotoxic CD8 T-cell responses (150). However, vaccination following infection and revaccination of hybrid-immune individuals resulted in a robust expansion of both cytotoxic CD4 and CD8 T-cell responses (117–121) (**Figure 3**) as well as Tfh levels (160, 161) that are likely key to robust affinity maturation and evolution of neutralizing antibody responses.

Additionally, unlike vaccination alone, hybrid immunity results in the induction of tissue-resident T-cell populations that can respond rapidly to infection (18, 19), pointing to a critical opportunity for revaccination to increase the number of effector cells at the site of viral replication. A study evaluating phenotype, specificity, function, and persistence of nasal-resident SARS-CoV-2–specific T cells showed almost exclusive detection of SARS-CoV-2–specific CD4 and CD8 T cells in nasal mucosa among individuals with hybrid immunity versus those with only vaccine-induced immunity (18), with nasal-resident T-cell responses persisting for ≥140 days post infection (18). Differences observed in hybrid immunity compared with vaccine-only immunity may be related to critical signals delivered to T cells during SARS-CoV-2 infection within the mucosa, which may be key to retaining T cells at the site of infection. Along these lines, preclinical studies have suggested that, following infection, nasal-associated lymphoid aggregates remain active in the tissue, supporting the persistence of virus-specific T cells (18, 165), particularly in the lower respiratory tract (105). Because memory T cells perpetually survey tissues, revaccination is likely to bolster both systemic and tissue-resident immunity, amplifying the number of effector cells able to respond to infection.

Both infection- and vaccine-induced T cells exhibit broad cross-reactivity against newly emerging variants (86), including XBB.1, BA.2.86, and beyond (87). Interestingly, the order of vaccination/infection appears to affect the CD8 T-cell response (166). Additionally, the frequency and functional diversity of T cells expands with successive doses of mRNA vaccination in a hybrid-immune population (121). Importantly, these expanded CD4 and CD8 T-cell responses lead to increased viral control and clearance (159).

In addition to enhanced humoral responses, hybrid immunity is associated with robust and sustained cellular immune responses, particularly characterized by elevated interferon-gamma (IFN-γ) secretion by antigen-specific CD4+ and CD8+ T cells. These T-cell responses are often polyfunctional, co-expressing IFN-γ, IL-2, and TNF-α, and display greater breadth and magnitude than those elicited by vaccination or infection alone (135). Importantly, IFN-γ production remains detectable for several months following antigen exposure and appears to be less affected by Spike protein mutations, contributing to more durable and cross-variant protection in hybrid- immune individuals (167, 168). Moreover, evidence suggests that different cytokine profiles lead to various helper functions, with similarities across both vaccinated and hybrid individuals (169). This enhanced T cell–mediated immunity likely plays a critical role in limiting disease severity, especially in the context of emerging variants with partial escape from neutralizing antibodies.

Overall, revaccination significantly enhances T-cell responses following infection, particularly within the mucosal compartments, leading to increased numbers of both CD4 and CD8 T cells at the sites of viral replication. This augmentation is critical for vulnerable populations, as it improves the body’s ability to rapidly respond to and clear infections, including by highly mutated SARS-CoV-2 variants. Thus, boosting not only strengthens the overall T-cell–mediated immune response but also ensures a robust and versatile defense mechanism against emerging variants of concern, as T-cell epitopes are more conserved across viral variants (170).

### 2.4 Revaccination as a mechanism to improve immune durability

The benefits of revaccination in hybrid-immune populations over time was modeled in the setting of individuals living in prison settings (171). Specifically, the impact of infection alone, vaccination alone, hybrid immunity, and revaccination on reducing the risk of infection in their close contacts was assessed (171). While hybrid immunity provided the greatest and most durable level of indirect protection, additional vaccine doses, especially those targeting circulating variants, provided additive benefits in those with infection-acquired immunity (171). Whether this enhanced durability of protection was due to characteristics of the infecting variant, improved quality of the immune response, or simply higher levels of antibody titers or T-cell frequencies remains incompletely defined.

Vaccination-induced immunity, while initially highly effective in limiting COVID-19 (172), clearly wanes over time, both due to decreasing systemic antibody concentrations but also due to the emergence of viral variants (132, 173). Importantly, the same trend of waning immunity was observed with infection-induced immunity, which has been shown to initially provide a high degree of protection against reinfection, but over time provides incomplete protection against emerging SARS-CoV-2 variants (174). Specifically, titers following vaccine-induced immunity have been shown to be more durable than those following infection, particularly with mRNA-based COVID-19 vaccines, with a median duration of 29.6 months (175). In comparison, infection offered a substantial initial immune response, but with a shorter median durability of 21.5 months (175). Hybrid immunity, resulting from infection followed by vaccination, shows enhanced and more durable immune responses than either vaccination or infection alone, leading to higher antibody titers and better cross-reactivity against different variants (176, 177). However, strikingly, following a third vaccine dose, waning declined, and after a fourth dose antibody titers remained remarkably stable (173). Furthermore, boosting of individuals with hybrid immunity exhibited more durable protection from reinfection (39), both in the pre-omicron and post-omicron era, suggesting that COVID-19 mRNA vaccination induces long-lasting transmission-blocking activity (178).

Beyond the effects of high-titer antibodies that provide a first line of defense against an incoming infection, the anamnestic immune response has been linked to long-term protection against COVID-19 (179). Higher frequencies of the circulating memory T and B cells that survey for infection increase the probability of a rapid and effective response to infection (152, 180, 181). Along these lines, the speed of the anamnestic antibody response was a critical predictor of viral control 1 year after mRNA vaccination in a non-human primate model (182). Importantly, the speed of the humoral, but not the T cell, response was a key determinant of viral load control in this preclinical model (182). Similarly, a rapid immune response upon reinfection with SARS-CoV-2 has been shown to be improved with hybrid immunity compared with vaccination alone in humans (22, 117), potentially via the rapid generation of germinal centers able to quickly adapt to the incoming variant resulting in accelerated generation of antibody-secreting cells producing up-to-date RBD-specific antibodies (22). Moreover, revaccination, as described above, increases both the frequency and quality of memory B cells and T cells, providing the potential for effective immune recall of diverse and high-quality memory cells in response to reinfection (16, 24, 82, 178). These data point to 2 potential mechanisms underlying durable immunity: 1) the persistence of a strong initial defense against the virus, characterized by sustained antibody titers and effector T cells, and 2) the maintenance of an expanded pool of memory B and T cells that can be quickly recalled. These memory cells are capable of rapidly recognizing and adapting to sequence changes, as well as proliferating in response to viral exposure with the capacity to traffic and respond rapidly at the site of infection. Routine revaccination is likely a key mechanism by which both of these lines of defense may be bolstered to provide both a first and second line of defense against SARS-CoV-2.

Among immunocompromised individuals, dampened antibody responses following SARS-CoV-2 infection and vaccination have been well established. Immunocompromised individuals often required multiple vaccine doses (up to 4 or 5) to achieve antibody titers comparable to those of immunocompetent individuals (183). Revaccination after a SARS-CoV-2 infection offers substantial benefits to these individuals by significantly enhancing both humoral and cellular immune responses (183). The combination of infection and vaccination has been shown to lead to a significant increase in neutralization capacity and cross-protection against emerging variants, including omicron variants, which have been more challenging due to their antigenic drift from the original virus strain (184). T-cell activation and memory T-cell formation were also stronger following revaccination in this group, providing a more robust and long-term immune defense against SARS-CoV-2 (158, 185). Moreover, this improved immune response translated into more favorable clinical outcomes for immunocompromised individuals with hybrid immunity compared with those who did not receive a vaccine post-infection, where individuals with hybrid immunity showed lower hospitalization rates and a reduced risk of severe COVID-19 complications (186). Yet, revaccination of immunocompromised populations with hybrid immunity was shown to update and replenish rapidly waning humoral immune responses (187), suggesting that such revaccination may be key to ensuring that these individuals experience mild or moderate symptoms when subsequently exposed to SARS-CoV-2.

### 2.5 Revaccination to prevent post-acute sequelae of COVID-19

In addition to acute phase outcomes, it is important to consider the post-acute sequelae of COVID-19. Long COVID, or post-acute sequelae of COVID-19, includes a myriad of complications affecting multiple organ systems, which, beyond 30 days after COVID-19 diagnosis, cause a substantial burden of health loss and increased risk of mortality (12). Findings from a cohort study utilizing electronic health record databases in the United States showed that risk of post-acute sequelae and death were substantially lower in individuals with hybrid immunity versus those with infection alone (188). Similarly, findings from a prospective study showed that unvaccinated individuals reported higher rates of post-acute sequelae at 6 months compared with those who were vaccinated (45.2% vs. 33.3%; p = 0.018) (189). However, additional doses of vaccine in those with hybrid immunity was shown to provide a protective effect in a large study of over 109,000 individuals in Germany, with a direct protective effect against post-COVID condition observed following a fourth vaccination (190).

Several hypotheses have been raised to explain the etiology of long COVID, including the potential persistence of viral reservoirs in specific organs/tissues that may lead to persistent inflammation and tissue damage (191, 192). Additionally, the association of long COVID with the emergence of pathological immune responses following resolution of acute infection remains a critical area of research in the field. However, the ability to improve immune surveillance, across all organs and tissues, to both prevent an initial acute tissue pathological insult or to contribute to persistent surveillance and elimination of reservoirs likely could play an important role in limiting long COVID. Vaccination after recovery from COVID-19 boosts the immune response (39), potentially helping to clear any residual virus and preventing its reactivation, which could contribute to prolonged symptoms. Thus, the expanded T- and B-cell responses observed with revaccination, in addition to hybrid immunity, may offer a critical means to not only provide protection against the acute inflammatory consequences of COVID-19, but also provide an additional defense against post-acute consequence of infection (121, 189).

## 3 Discussion

The global SARS-CoV-2–specific immune landscape has progressed to one that is largely hybrid immune (2–4). However, hybrid immunity is highly heterogenous due to exposure to different variants and differences in vaccination history, as well as differences in levels of natural antigen exposure. Conversely, annual revaccination robustly raises immunity across all populations, bolstering humoral and cellular immunity irrespective of infection and vaccination history and thereby providing protective immunity across vaccinees. Yet, revaccination with variant-updated vaccines also provides critical value, inducing an immune response to protect against novel SARS-CoV-2 variants, reinforcing the need for updated booster vaccines as new variants of concern emerge (5). While there is no distinct seasonality to COVID-19, similar to other respiratory viruses, peaks are typically seen in the winter (50). As such, preseason boosting can play a critical role in harmonizing the magnitude, breadth, and contemporaneousness of the immune response, equipping revaccinated individuals with more effective immunity when there is a higher probability of exposure to the virus. While immune imprinting provides a critical evolutionary mechanism to rapidly adapt and expand a new antibody-secreting response from memory B cells, the narrower range of potential B-cell responses may incompletely explore the potential landscape of more potent humoral immune responses that may provide the highest level of protection (53). Thus, while vaccine design is underway to define novel antigen design approaches to overcome imprinting, boosting with contemporaneous variants provides a means to rapidly update a memory B-cell population and create a broader repertoire of clones. Although this repertoire may not be perfectly matched to the next strain that may emerge, it provides a mechanism to shift the response to accumulating mutations in the variant evolutionary landscape. Additionally, revaccination may increase the population of tissue-resident effector T cells, which are crucial for a rapid and timely response to infection (18, 19). As such, updated variant–containing booster vaccines are needed to avoid perpetual recall of archived immune responses, and instead enable preexisting immunity to adapt and provide optimal protection against newly circulating strains. Accordingly, global recommending bodies have begun to harmonize COVID-19 vaccine composition to more closely match the predominantly circulating variants and simplify the vaccine schedules (8, 36). Monovalent variant–updated vaccines are currently recommended to continue to shift the humoral immune response forward and avoid back-boosting, to protect against the currently circulating sublineages (36).

Future strategies should consider the prospect of blocking transmission of SARS-CoV-2 by modulating immune responses in the respiratory mucosa to provide localized protection and recall responses at the sites of viral entry (193). However, given that a vast majority of the population has hybrid immunity with both systemic and mucosal immune responses to SARS-CoV-2, repeated intramuscular vaccination may be sufficient for continued protection from COVID-19. A more thorough understanding of the extent and duration of protection against reinfection through hybrid immunity is crucial for continued public health planning, as such information may guide recommendations for optimal COVID-19 vaccine timing following SARS-CoV-2 infection (66).

Although hybrid immunity confers broader and more durable immune protection than either modality alone, it is increasingly challenged by the rapid antigenic evolution of SARS-CoV-2. Variants such as XBB.1.5, EG.5, and JN.1 exhibit substantial immune escape from neutralizing antibodies generated by previous exposures, even in individuals with hybrid immunity (107, 194). While cellular responses (e.g., memory T cells) provide protection against severe disease, these responses alone do not prevent infection or transmission, and the growing frequency of reinfections illustrates this limitation, highlighting the need for continued vaccination against the current variants.

Epidemiological data show that reinfections are rising in frequency and occurring at shorter intervals, with some individuals experiencing three or more infections within a two-year span, particularly during periods of Omicron subvariant dominance (195, 196). Furthermore, while prior immunity continues to reduce the risk of hospitalization and death, susceptibility to symptomatic infection remains significant with each new wave of antigenically distinct variants (197). In hybrid-immune populations, susceptibility to symptomatic reinfection persists, particularly among older adults, immunocompromised individuals, and those with comorbidities (25). This highlights the critical limitation of relying solely on existing immunity shaped by outdated antigen exposures.

This ongoing pattern of reinfection underscores a critical problem: current vaccine-induced and infection-induced immunity is often based on outdated antigenic exposures. Immune imprinting—where immune memory preferentially targets epitopes from the original viral strain—may further limit the effectiveness of responses to new variants (198). Re-vaccination with updated formulations is therefore essential to realign the immune response to the circulating virus. In the context of hybrid immunity, re-vaccination can amplify and refocus immune memory, mitigating the risk of breakthrough infections and potentially curbing onward transmission. Revaccination in hybrid-immune individuals drives the maturation and expansion of neutralizing and non-neutralizing antibodies, and enhancing mucosal and tissue-resident immunity. It facilitates rapid adaptation of pre-existing memory B cells, promotes the development of broader and more variant-resilient antibody repertoires, and boosts mucosal IgA and effector T-cell responses at key sites of viral entry. Importantly, updated boosters have been shown to improve protection against highly immune-evasive strains such as XBB.1.5 and KP.2 (72, 199). Revaccination also improves the durability of immune protection, enhances immune responses in immunocompromised individuals, and reduces the risk of post-acute sequelae such as long COVID (12, 16, 81). Thus, in the context of an ever-shifting viral landscape, revaccination with updated formulations is not only a mechanism to restore and broaden protection but also a critical tool to reduce infection, transmission, severe disease, and long-term complications in both general and vulnerable populations.
